# Evolving Musical Sight Reading Exercises Using Expert Models

**DOI:** 10.3389/frai.2020.497530

**Published:** 2021-01-27

**Authors:** Charlotte Pierce, Tim Hendtlass, Anthony Bartel, Clinton J. Woodward

**Affiliations:** ^1^ Melbourne School of Engineering, The University of Melbourne, Parkville, VI, Australia; ^2^ Faculty of Science, Engineering and Technology, Swinburne University of Technology, Hawthorn, VI, Australia; ^3^ Faculty of Health, Arts and Design, Swinburne University of Technology, Hawthorn, VI, Australia

**Keywords:** expert models, musical sight reading, melody representation, music education, evolutionary algorithms

## Abstract

Sight reading skills are widely considered to be crucial for all musicians. However, given that sight reading involves playing sheet music without having seen it before, once an exercise has been completed by a student it can no longer be used as a sight reading exercise for them. In this paper we present a novel evolutionary algorithm for generating musical sight reading exercises in the Western art music tradition. Using models based on expert examples, the algorithm generates material suitable for practice which is both technically appropriate and aesthetically pleasing with respect to an instrument and difficulty level. This overcomes the resource constraint in using traditional practice exercises, which are exhausted quickly by students and teachers due to their limited quantity.

## 1 Introduction

Sight reading is widely believed to be a basic skill every musician should obtain (Spillman, 1990; Crozier, 2000; Lehmann and McArthur, 2002; McPherson and Gabrielsson, 2002; Galyen, 2005; Kopiez and In Lee, 2008), and is required at most levels of formal musical achievement in many countries (Ji, 2017; Australian Music Examinations Board, 2018a; The Associated Board of the Royal Schools of Music, 2018). It enables musicians to learn new music quickly, to rapidly expose themselves to a variety of repertoire and musical styles, and to become independent musical learners (Gregory, 1972). For students specifically, good sight reading skills allow them to dedicate more lesson time to musical interpretation rather than learning notes. For music teachers, sight reading is essential for demonstrating examples to their students.

As with most skills, practice is key to improving musical sight reading ability. This is shown by Kopiez and In Lee (2008), who found that there is a positive correlation between the time a person has spent practicing sight reading and their level of sight reading skill. As sight reading is the ability to perform a piece or phrase of music without having seen it before, as soon as a single exercise has been completed once by an individual it is no longer a sight reading exercise for them (Schulz, 2016). Currently, practice material for students preparing for formal music exams in the Western tradition is written by experts and disseminated to students through online stores and physical books. This is an ineffective approach. Access to expertize is limited, and practice material is consumed much faster than it is created. This means that students often exhaust the available resources before achieving competency. Given that both the quantity and quality of practice is key to gaining competence in sight reading (Kornicke, 1992; Banton, 1995; Galyen, 2005; Kopiez and In Lee, 2008; Tsangari, 2010), this resource constraint is a large barrier for musicians attempting to develop the skill.

To overcome this resource constraint, in this paper we present a novel evolutionary algorithm (EA) for generating monophonic sight reading exercises in the Western art music tradition. The goal of the algorithm is to generate exercises which are both technically appropriate and aesthetically pleasing. It does so by using expert models of professionally-written sight reading exercises as templates for emulation.

There are four primary reasons for selecting EAs in this work. First, Biles (2007) notes that evolutionary approaches have been applied to melody generation problems more often than any other technique, and with more success. Secondly, the solution space when generating a melody is large, and evolutionary algorithms are well suited to navigating that space (Johnson et al., 2004). Thirdly, evolutionary algorithms allow for specific goals to be set for a solution while still providing space for random elements and emergent behaviors to appear. This means that the solutions found by the algorithm are likely to maintain a higher level of variability compared to other methods, even when using identical configurations. Lastly, preliminary experiments using probability-bound random sampling indicated that simpler approaches were not able to satisfactorily handle the complexities of the problem.

**FIGURE 1:**
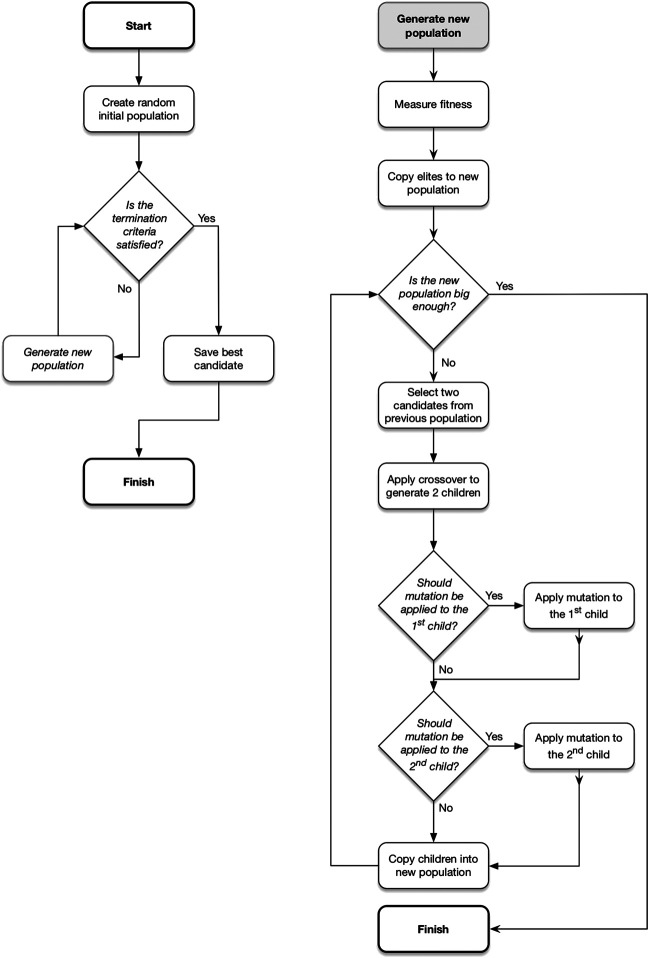
The general process followed by an evolutionary algorithm. Recreated from Koza (2018). Note that the number of parent candidates selected and the number of children generated depends on the operator. This example shows two parents generating two offspring.

Figure 1 shows the general process followed by EAs. An EA begins with a randomly generated set of candidate solutions, referred to as a ‘population’, then follows an iterative process until some termination criteria is met. This iterative process involves generating a series of new candidate populations, each of which is based on the previous. The aim is that over time the populations will contain incrementally superior solutions to those in previous populations.

First, all candidates are measured for their suitability as a solution and assigned a corresponding numerical value (i.e., fitness value). The top *n* best or “elite” candidates (where *n* can be 0) are then directly copied into the next population without any alterations. The remainder of the new population is formed through the application of the genetic operators *crossover* and *mutation*. Crossover is a reproduction method used to create candidates by combining elements from two ‘parents’ from the previous population. The mutation operator applies small random changes to a candidate in order to introduce diversity into the population.

Once the new population has reached its target size, the termination criteria are checked. If they have been met, the candidate with the highest fitness over all iterations is returned as the solution. If not, the process is repeated.

The algorithm requires a number of aspects be defined:

- **Population size** The number of candidate solutions in a population. If this value is too small the algorithm may converge on a suboptimal solution due to lack of diversity. However, if this value is too large the algorithm may take an excessive amount of time to finish.
- **Termination criteria** When the algorithm should stop. It is typically a target fitness value, a specific number of iterations, a number of iterations without improvement, or a combination of the three. For example, target a fitness of 0.95, but if it hasn’t been reached within 1,000 iterations terminate the algorithm anyway.
- **Fitness function** A numerical measure for quantifying the suitability of a candidate solution. This dictates the likelihood that a candidate will be selected to be part of the next population.
- **Number of elites** The number of top candidates from the previous population that will be directly copied to the new population without any adjustments.
- **Genetic operators** How the *crossover* and *mutation* operators will be implemented.
- **Selection method** The method for selecting candidates for the crossover operator. Typically a function of each candidate’s fitness value.
- **Probability of mutation** How likely it is that candidates resulting from the crossover operator will be mutated.
- **Candidate representation** How each candidate is encoded.

Section 2 will describe the method used in this work. This includes the curation of a suitable set of expert models, the technique used to represent candidate solutions, and the experimental design. The results of this experimental design will be detailed in Section 3. Finally, Section 4 will discuss the implications of these results and potential directions for future work.

## 2 Method

### 2.1 Building Expert Models

Four books of sight reading exercises were selected, representative of the curricula of the Australian Music Examinations Board (AMEB), Associated Board of the Royal Schools of Music (ABRSM), and Trinity College. Grade 1 and 2 exercises were extracted from each book, as summarized in Table 1. A expert model was derived from each exercise, capturing the following characteristics:

- Key and time signatures
- length,
- range,
- number of ties and rests
- ratio of notes to rests
- proportions of note lengths, rest lengths, and intervals, and
- melody shape (defined in Section 2.3.2).

**TABLE 1:** Summary of expert-written example exercises extracted from published books.

Book	Grade 1	Grade 2
Improve your sight-reading! (Harris, 1994)	25	16
Flute sight-reading (Selleck, 2012)	12	12
Sound at sight - sight reading pieces for flute; book 1 (Rae, 2007)	20	20
Flute specimen sight reading	15	16
The Associated Board of the Royal Schools of Music (1995)		
**Total**	72	64

These characteristics can be viewed as a whole to gain an appreciation of a “typical” exercise at the Grade 1 and 2 difficulty levels. They can also be considered individually to see a distilled view of the key characteristics of each individual exercise. In practice, this data will primarily be used at the level of an individual exercise, where the set of characteristics relating to one exercise is used to form a single expert model. This is discussed further in Section 2.4.1.

### 2.2 Exercise Representation

Many published works in the field of melody generation are not clear on how melodies are represented. However, two primary themes emerge: tree-based and sequential structures (Biles, 2007). This is true for works which both do and do not utilize evolutionary algorithms.

Sequential structures, such as that used by Acevedo (2004), represent melodies as ordered lists of musical elements. These elements are typically individual notes and rests, with each having a length and (where appropriate) a pitch. Pitches can be represented absolutely (e.g., C4), or as an offset from some epoch. Length can also be represented absolutely (e.g., crotchet), or as a start and end time offset from the start of the melody. In some cases, elements may also contain ornamental information such as dynamic or articulation markings. Melodies represented this way are read by examining the sequence of musical elements in order.

Regardless of the specific encoding scheme, sequential structures are not an ideal choice for an evolutionary algorithm. This is particularly true in this work, where the desired result is a melody of an explicit, fixed length. The practical reason for this lies in the crossover operator. This operator swaps sections of two parents to create two new candidates. When using a sequential structure, it is easy for the newly created candidates to have different lengths, simply by choosing asymmetrical crossover points. It is also easy for crossover to create candidates whose note and rest sequences do not fit neatly into entire bar lengths. For example, if two 4-bar parents were selected, while asymmetrical crossover could result in two children with 4-bar lengths it is much more likely to create two children with different lengths and partially complete bars (e.g., 3.4, and 4.6 bars). Symmetrical crossover would eliminate this problem, but would severely reduce the variety of new candidates that could be created, as the selected crossover points would always need to be symmetrical.

While not without fault, tree-based structures avoid these problems entirely. As such, they are used in this work.

#### 2.2.1 Tree-Based Structures in the Literature

**FIGURE 2:**
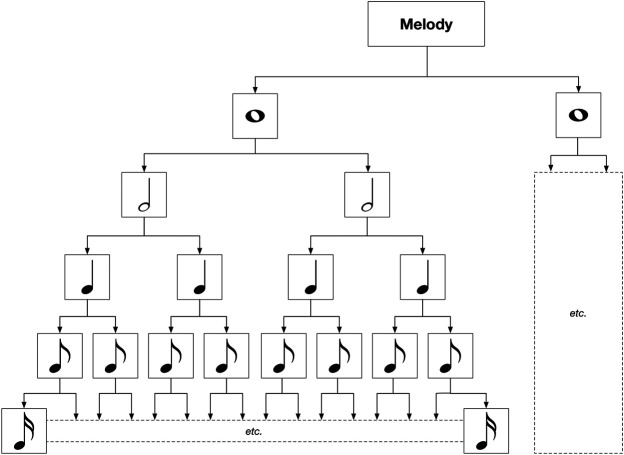
The duration hierarchy typically used by tree structures which are designed to represent melodies in common time. The bottom row of semiquavers has been truncated for space, and the right-hand side of the tree is not shown to completion as it is identical to the left.

In the literature, tree-based solutions for melody representation typically follow a binary structure where each node represents a musical element with half the duration of its parent. This means that the structure of a melody tree adheres to the duration hierarchy shown in Figure 2, where the length of a note is entirely dependent on its depth within the tree. The root of the tree represents the entire melody, with nodes in the first layer representing individual bars. From this point onwards each additional layer of depth splits durations in two. For example, in a $\frac{4}{4}$ melody nodes with a depth of 1 would represent semibreves, nodes with a depth of 2 would represent minims, nodes with a depth of 3 would represent crotchets, and so on. Within this structure only leaf nodes represent concrete musical elements that would be directly shown on a score. Internal nodes, at a minimum, serve to maintain the duration hierarchy. However, some implementations also assign some or all internal nodes special meaning in order to support additional functionality. When interpreting a melody tree, leaf nodes on the left are typically played before leaf nodes on the right.

Table 2 shows a comparison of the characteristics of the melody trees in the literature. All of the trees are able to represent monophonic melodies and dotted notes. Additionally, all three representations allow for subtrees from two different melodies to be swapped at any point, without breaking the tree structure.

**TABLE 2:** A comparison of the features of the melody trees proposed in the literature.

Feature	Rizo et al. (2003)	Dahlstedt (2007)	de León et al. (2016)
Monophony	✓	✓	✓
Polyphony	✗	✓	✗
Dotted notes	✓	✓	✓
Tied notes	✗	✓	✓
Irregular divisions	✗	✗	✗
Simple time	✓	✗	✓
Compound time	✗	✗	✗
Maintain musical grammar	✓	✗	✓
Crossover anywhere	✓	✓	✓

The trees proposed by Rizo et al. (2003) and de León et al. (2016) both support simple time signatures–that is, time signatures such as $\frac{4}{4}$ and $\frac{2}{4}$ where measures can recursively be divided into equal halves without the need for dotted notes. However, neither of these trees support compound time signatures such as $\frac{6}{8}$ and $\frac{9}{8}$, where measures do not neatly fit into a binary structure. They also do not support simple time signatures such as $\frac{3}{4}$ which do not neatly divide into two.

Dahlstedt’s tree is noted as supporting neither simple nor compound time. This is because the tree does not structure its nodes according to a duration hierarchy. Instead, it assigns the duration of nodes independently of one another. A time signature is applied to the melody when translating it to a score rather than within the tree itself, and the melody has no guarantee of fitting within the chosen time signature. This means that Dahlstedt’s tree, unlike the trees of Rizo et al. (2003) and de León et al. (2016), also does not maintain a musical grammar. That is, it can not guarantee that the represented melody will fit neatly into any particular time signature.

None of the melody tree structures discussed explicitly support irregular divisions such as triplets or tuplets. This severely limits their representational capacities. Rizo’s tree has an additional problem, in that it does not offer a mechanism for representing tied notes.

For representing sight reading exercises a melody tree would, at a minimum, need to support:

- monophonic melodies
- dotted notes
- tied notes
- triplets
- simple time signatures
- compound time signatures,
- enforceable musical grammar, and
- swapping of subtrees at any point.

Additional features that would be useful for representing melodies include support for

- polyphonic melodies
- multiple time signatures in one melody
- ornamental and stylistic markings (e.g., mordents, trills), and
- additional irregular divisions (e.g., duplets, any variation of the “*x* in the time of *y*” pattern).

These additional features are not necessary for the task of generating monophonic sight reading melodies of low level difficulties, and thus are left as future work.

None of the trees in the surveyed literature support the necessary combination of minimally viable features. As such, a novel melody tree was created that would meet this criteria. This novel tree is described in Section 2.2.2.

#### 2.2.2 Designing a Novel Melody Tree

Several of the minimally viable features for a melody tree are already supported in existing trees. This is capitalized upon in this work by taking elements from existing trees where possible then adding the additional, missing functionality necessary for representing musical sight reading exercises.

Of the trees in the literature, that proposed by Rizo et al. (2003) offers the most desired features, thus will act as a starting point for a novel tree structure. The features covered by this tree include support for:

- monophonic melodies
- dotted notes
- tied notes
- simple time signatures,
- enforceable musical grammar, and
- swapping subtrees at any point.

This leaves two key features absent:

- upport for compound time signatures
- Support for triplets

The implementation of these two features is discussed below.

##### 2.2.2.1 Supporting Compound Time Signatures

The ability to swap subtrees at any point while enforcing musical grammar is entirely due to a tree following a duration hierarchy as described in Section 2.2.1. Unfortunately, this encourages a binary structure, which is not ideal for representing compound time signatures or other time signatures which do not neatly divide into two. For example, to represent a melody in $\frac{3}{4}$ time (a simple time signature that does not neatly divide into two), splitting bars equally would result in the first layer of nodes representing dotted crotchets. Continuing this pattern, the following layer would contain nodes representing half that value again–a dotted quaver. The next layer would represent dotted semiquavers, then dotted demisemiquavers, and so on. This pattern results in a structure where node lengths are unnecessarily complex, and individual nodes do not represent lengths commonly found in melodies (i.e., non-dotted lengths).

An alternative strategy might be to split any compound-lengthed node into two non-equal but more typical lengths. Returning to the example of a melody in $\frac{3}{4}$ time, this would result in the first layer being a combination of a minim and crotchet node. The next layer would then comprise two crotchet nodes (from splitting the minim) and two quaver nodes (from splitting the crotchet).

This approach presents two problems. Firstly, it breaks the duration hierarchy which requires that all nodes at the same depth have the same length. This complicates the continuation operator as no assumptions can be made regarding a node’s length with respect to its depth. It also adds more complexity when swapping subtrees in ensuring nodes are reassigned the correct length given their new depths.

Secondly, it introduces a decision regarding which node should be left-most in the tree–the longer or shorter of the split? For example, when splitting a $\frac{3}{4}$ bar should the minim or crotchet node be left-most? This choice informs how elegantly a melody can be represented and how often continuation operators need to be used to form longer note lengths.

The solution to these problems is for bars of a time signature $\frac{n}{m}$ be split into *n* nodes of *m* length, where *m* indicates the number of that length note required to equal the length of a semibreve. So, *m* = 1 indicates a semibreve, *m* = 2 indicates a minim, *m* = 4 indicates a crotchet, and so on.

This strategy means that the second layer of a melody tree is non-binary, but the remainder is. Unfortunately, this gives rise to one problem. In order to successfully implement the crossover operator, it must be possible to swap any subtree from one melody with any subtree from another. This is an issue when the second layer of the tree is non-binary, as the parent node of a non-binary layer (i.e., the node representing a single bar) may be swapped with the parent node of a binary layer.

The solution to this problem is to remove the layer of ‘bar’ nodes entirely, meaning that the tree starts with a layer containing n* *number*_*of*_*bar* nodes of *m* length. This means that the first layer of the tree may contain many nodes, but every one of those nodes is binary and has children with exactly half of their length. Additionally, because the value of ‘m’ is taken from the time signature, these nodes are guaranteed to be of a length which can be recursively split into two equal, non-compound halves. Notes longer than *m* can still be represented through the use of one or several linked continuation operators.

2.2.2.2 Supporting Triplets.

**FIGURE 3:**
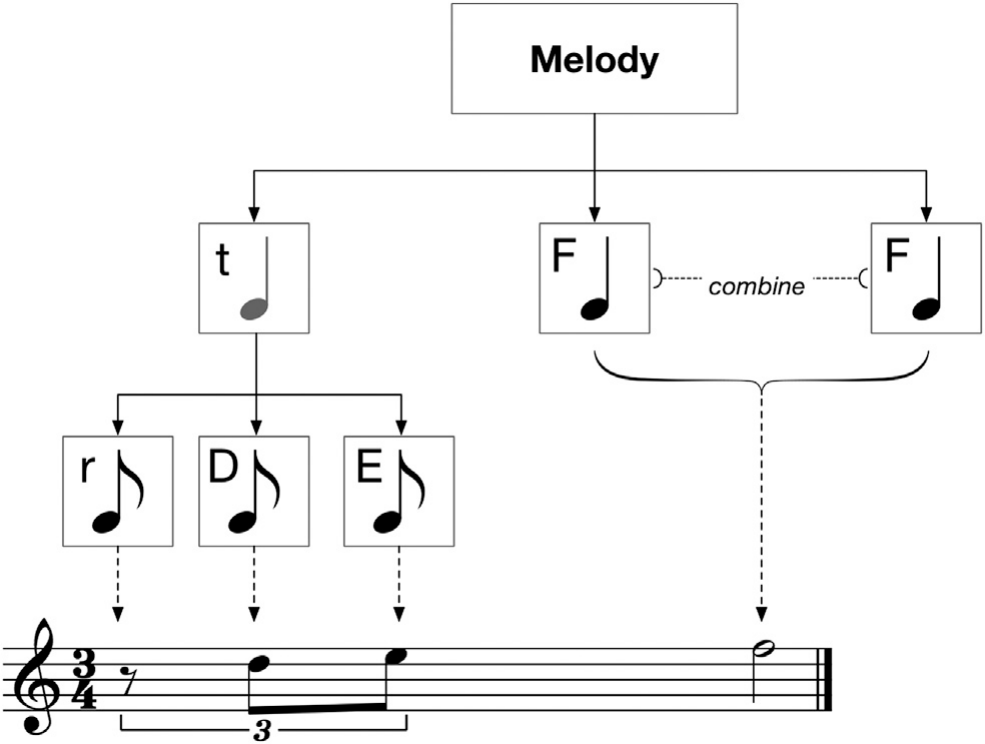
A melody tree containing a triplet and a combine operator.

Triplets are implemented with an internal node operator similar to the *split* and *continuation* operators used by de León et al. (2016). As shown in Figure 3, the triplet operator is placed on the first direct parent of the triplet leaf nodes. If all leaves within the triplet are of the same length, the triplet operator is placed one layer above. If the leaves within the triplet are of different lengths, the triplet operator is placed on the first common parent.

The triplet operator does not break the duration hierarchy, nor does it restrict the swapping of subtrees. If the triplet operator itself is selected to be swapped, the entire triplet is moved. If a subtree within the triplet is selected to swap, notes within that subtree will–assuming they are swapped to a non-triplet parent node–be interpreted as having a standard, non-triplet length. Conversely, the subtree swapped into its place will then be interpreted as part of the triplet.

### 2.3 Evolutionary Operators

#### 2.3.1 Parent Selection

In this work, Pareto selection is used. Instead of considering a candidate’s overall fitness value, Pareto selection examines fitness in terms of individual characteristics. This is a relative probability measure which is useful for situations where a single fitness value does not make sense (Horn et al., 1994; Fonseca and Fleming, 1995).

For example, consider the task of evolving a box with an appropriate width, depth, height, strength, and weight. Here, an overall or combined fitness value will not work, as perfection in one aspect of the box does not offset weakness in another aspect. That is, better fitness in height does not compensate for poor fitness in depth. Similarly, good fitness in width does not make up for poor fitness in strength. Pareto selection deals with this issue by considering the individual aspects of fitness. The probability that a candidate will be selected is based on the number of other candidates in the same population that it is superior to in every aspect. Using the box example, a candidate is only better than another candidate if it has a superior width, depth, height, strength, and weight. Once this value is known, Eq. 1 can be used to determine the selection probability for a candidate.

$$\begin{array}{l} probability\_of\_selection_{i}=\frac{\left(1+W_{i}\right)}{{\sum^{\text{​}}}_{j=1}^{n}W_{j}}\\ W_{i}:\text{the number of candidates the }i^{th}\text{ candidate in a population}\\ \;\;\;\;\;\;\;\text{is superior to in every aspect}\\ n:\text{the total number of candidates in the population} \end{array}$$ (1)

The task of evolving musical sight reading exercises benefits from the use of Pareto selection. An exercise needs to meet multiple criteria, both technical and esthetic. For example, an exercise at the Grade 1 level might need to use only crotchet lengths and have only one or two rests. Additionally, the melody might also need to meet esthetic criteria such as beginning and ending on the tonic note. As with the box problem, an overall fitness value does not work for these requirements. A good selection of note lengths does not make up for a lack of esthetic qualities. Similarly, a melody sounding good does not make up for an absence of appropriate technical characteristics. As such, Pareto selection is an ideal solution.

#### 2.3.2 Fitness Measures

The fitness measures are designed to guide the evolutionary process toward creating a melody with a specific set of characteristics. For this work six measures are used, each of which has an associated target value. A melody is assigned a score in the range [0..1] for each measure. This score is calculated using Eq. 2 as the difference between the candidate’s actual and target value for a fitness measure.

$$\begin{array}{l} fitness_{fi}=1.0-abs\left(t_{f}-a_{fi}\right)\\ t_{f}:\text{the target value for fitness measure }f\\ a_{fi}:\text{the actual value for fitness measure }f\text{ for candidate }i \end{array}$$ (2)

To illustrate this idea, return to the example of evolving a box. A target height for the box may be set as 10 cm. If a candidate box had a height of 10 cm it would receive a score of 1.0 for the ‘height’ measure. However, if the box had a height of 5 cm it would receive a score of 0.5. Similarly, if the box overshot the target with a height of 15 cm it would also receive a score of 0.5.

Each of the six fitness measures used in this work are based on counting a specific element within the melody. These counts are described as being either “time” or “count” based. A count-based measure takes the count as a raw value. For example, 6 notes in the melody are crotchets. Time-based measures take the raw count value and interpret it as a proportion of melody time. For example, in a 4 bar melody in $\frac{4}{4}$ time a count of 8 crotchets would be interpreted as 50% of the melody being crotchets, because the melody could potentially fit a total of 16 crotchets.

The six fitness measures used in this work are:

- **Target note lengths (time-based)** The proportion of melody time to be taken by each note length. For example, 50% of the melody time should be filled by crotchets; 25% of the melody time should be filled by quavers.
- **Target rest lengths (time-based)** The proportion of melody time to be taken by each rest length. For example, 25% of the melody time should be filled by crotchet rests.
- **Allowable lengths (count-based)** The acceptable lengths for notes and rests in the melody. For example, only use notes and rests with crotchet or quaver lengths.
- **Target intervals (count-based)** The proportion of each size of interval to include. Size is represented in scale degrees. For example, 50% of intervals should be 1 scale degree in size; 50% of intervals should be 2 scale degrees in size.
- **Allowable intervals (count-based)** The acceptable interval sizes to use in the melody. Size is represented in scale degrees. For example, only use intervals with sizes of 1 or 2 scale degrees.
- **Melody shape (count-based)** The number of segments in the melody containing three contiguous notes where the pitches move consistently up or down. For example, 80% of the melody segments should be shapely.

The target note proportions and target rest proportions should sum to represent exactly 100% of the melody time. Similarly, the target interval proportions should sum to represent 100% of the intervals. The allowable lengths and allowable intervals are derived automatically from the target note, rest, and interval proportions. For example, if target proportions are set for crotchet and quaver notes, and a target proportion is set for crotchet rests, the allowable lengths are crotchets and quavers. Similarly, if target proportions are set for intervals of size 1, 2, and 3, the allowable intervals are 1, 2, and 3.

The purpose of combining “target” and “allowable” measures rather than just using one or the other is to guide the algorithm toward rewarding the use of an “allowable” length more than an undesirable length, even if doing so breaks the target proportions. The “target” measures represent the ideal proportions of note lengths and intervals. However, if the algorithm is struggling to reach the ideal targets it is better to sacrifice melodies score with respect to the targets, in the interest of still only using “allowable” note lengths and intervals. This is because introducing “unallowable” note lengths or intervals is a much bigger problem for the overall fitness-for-purpose of an exercise than having slightly incorrect proportions. That is, if only crotchets and quavers are “allowable” and the algorithm can not form an exercise with the desired proportions of these note lengths, it is still better for the algorithm to use extra quavers and potentially lower the score for *target note proportions* than to introduce some other note length that is considered inappropriate.

**FIGURE 4:**
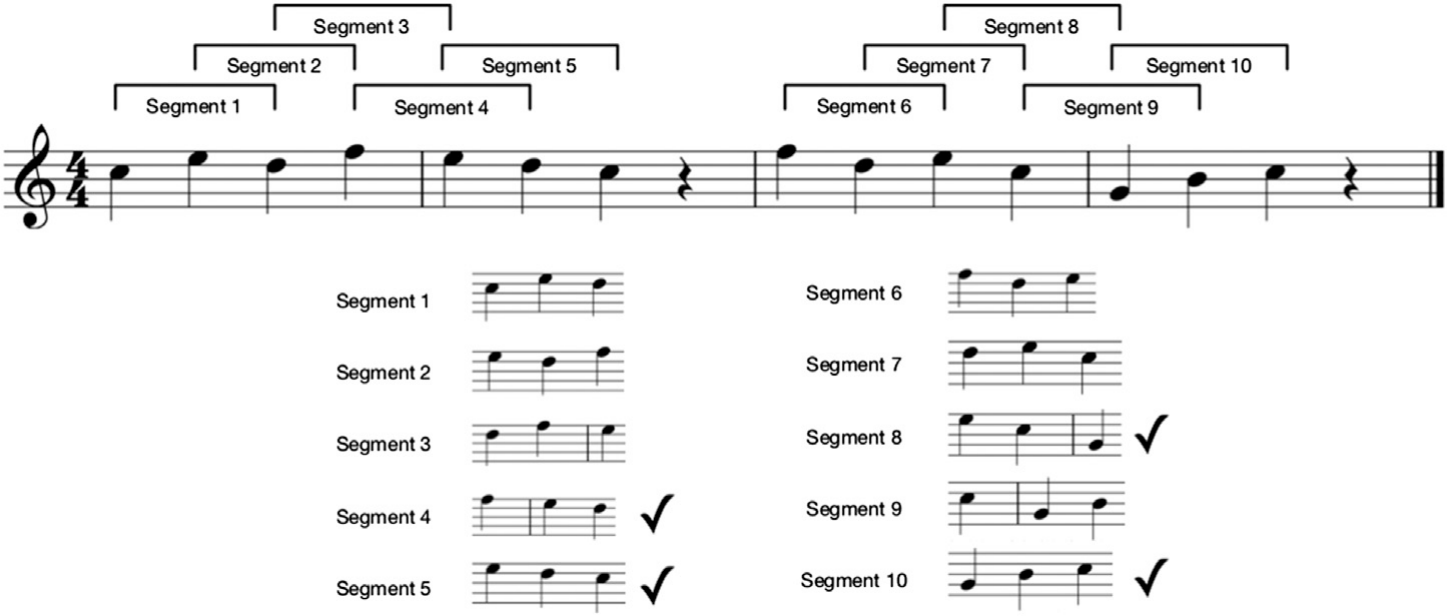
Calculating the shape of a melody. Tick marks indicate the segments which are counted as having *shape*, as the pitches within the segment move consistently up or down.

The “melody shape” measure is illustrated further in Figure 4. In this example, the melody contains a total of ten segments. Note that segments containing rests or fewer than three notes are not counted. Of these ten segments, only four contain notes which move consistently up or down in pitch. Therefore, in this case the melody shape is $\frac{4}{10}$, or 0.4.

Consider the example target values for a melody set out in Table 3. These targets indicate that the evolutionary algorithm should attempt to create a melody made entirely of crotchets, most of which are notes (as opposed to rests). They specify a large target proportion for intervals with a size of 1, but also requests that some intervals of 0 and 2 scale degrees be used. The melody shape target is set to 0.3, meaning that 30% of the segments in the melody should have notes which move consistently up or down in pitch.

**TABLE 3:** Calculating the fitness of the melody in Figure 5 against an arbitrarily selected set of targets. The final fitness value for each measure is in bold.

Fitness measure	Target	Actual	Fitness
Target note proportions	0.75 crotchets	Crotchets	$1-abs\left(0.75-\frac{13}{16}\right)=0\mathrm{.94}$
Target rest proportions	0.25 crotchets	$\frac{2}{16}$ crotchets	$1-abs\left(0.25-\frac{2}{16}\right)=0\mathrm{.88}$
Allowable lengths	Crotchets	$\frac{15}{17}$ allowable Lengths	$\frac{15}{17}\approx 0\mathrm{.88}$
Target interval	0.3 size 0	$\frac{0}{13}$ intervals Of size 0	1−abs(0.3−0)=0.7
Proportions	0.5 size 1	$\frac{9}{13}$ intervals Of size 1	$1-abs\left(0.5-\frac{9}{13}\right)\approx 0.81$
	0.2 size 2	$\frac{3}{13}$ intervals Of size 2	$1-abs\left(0.2-\frac{3}{13}\right)\approx 0.97$
			$\frac{\left(0.7+0.81+0.97\right)}{3}\approx 0\mathrm{.83}$
Allowable intervals	0, 1, and 2	$\frac{12}{13}$ allowable Intervals	$\frac{12}{13}\approx 0\mathrm{.92}$
Melody shape	0.3	$\frac{6}{11}$ shapely Segments	$1-abs\left(0.3-\frac{6}{11}\right)\approx 0\mathrm{.75}$

**FIGURE 5:**
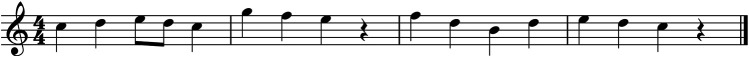
An example melody.

Now consider the melody shown in Figure 5. To calculate the fitness value for each measure we must calculate the actual proportions and numbers of notes, rests, and intervals, and compare them to the targets. For measures such as “target note/rest proportions”, “target interval proportions”, and “melody shape”, the fitness value is calculated according to Eq. 2. The remaining fitness measures are calculated as raw counts, as their target is for 100% of the melody to fit within the assigned parameters. For example, the fitness for “allowable intervals” is calculated as the number of intervals of an allowable size divided by the total number of intervals in the melody. Table 3 shows how each fitness measure would be calculated for this melody, given an arbitrarily selected set of targets.

#### 2.3.3 Crossover

The implementation of crossover is reasonably straightforward. First, two parent candidates are selected using Pareto selection. Then, a node from the melody tree of each candidate is randomly picked. The subtrees starting from these nodes are taken from each parent and their positions are swapped.

Once the subtrees are swapped, the durations of their nodes are altered with respect to their overall depth within their new tree. For example, if a node is placed one layer higher in its new tree than in its old tree, its duration is doubled. Similarly, a node placed one layer lower would have its duration halved. No additional alterations are made.

This simple implementation is possible because the melody tree representation ensures that no matter which subtrees are swapped the resulting melody trees will still be grammatically correct. Additionally, the length of the melodies remains fixed as the node durations are adjusted according to their new depths.

#### 2.3.4 Mutation

As the candidates in this work are melodies, the potential mutations are musical in nature and specific to the domain. One alteration type is randomly selected from the following:

- **Change note type** Randomly select a leaf node. If the node represents a note, change it to a rest. If the node represents a rest, change it to a note with a random pitch.
- **Split node** Randomly select a leaf node. Change that node into an internal node with two randomly initialized children.
- **Reduce node** Randomly select a leaf node. Remove that node and its siblings and randomly reinitialize their parent.
- **Reinitialize note** Randomly select a node representing a note (not a rest). Reinitialize the node with a random pitch.
- **Add triplet** Randomly select any node within the tree. If the node is a leaf, change it to an internal node, add the triplet operator, and randomly initialize and add three children to it. If the node already has children, add the triplet operator and randomly initialize and add a third child to it.
- **Remove triplet** Randomly select any node within the tree that has a triplet operator attached. Remove the triplet operator then randomly select one of the node’s children and remove the subtree from that point.
- **Add continuation** Randomly select any leaf node within the tree that does not already have a continuation operator attached. Add a continuation operator to the node. Then, if the next leaf node in the tree is not the same type, change it so that it is. For example, if the randomly selected node is a note and the next leaf node is a rest, change the rest to a note with the same pitch as the randomly selected node.
- **Remove continuation** Randomly select any node that has a continuation operator attached. Remove the operator.

Any change that is not possible is not considered when randomly selecting an alteration. For example, if a melody does not contain any triplets then “Remove triplet” will not be selected. The algorithm configuration also allows for individual mutation operators to be disabled regardless of whether they are possible or not for any given candidate. Additionally, the random elements of mutation (e.g., giving a note a new randomly selected pitch) are constrained with respect to a target range and key signature as dictated by an expert model.

### 2.4 Experimental Design

#### 2.4.1 Overview

The design of the algorithm allows for exercises to be generated for any monophonic instrument. However, for consistency and comparability between results only a single instrument–the flute–is used in this experimental design. The flute was chosen as it is both monophonic and non-transposing. It is also a relatively popular instrument, meaning there are a large number of sight reading exercise books published for it. This is important as parameter sets for the algorithm were derived from the characteristics of expert models, which are in turn derived from published books of exercises. This ensures that the targets set for the algorithm are both realistic and grounded in accepted, widely-used, professionally written examples.

Grades 1 and 2 were chosen as the difficulty levels with which to validate the algorithm’s capabilities. The reason for selecting these earlier grades lies in the utility of the exercises. Formal exams at the Grade 1 level are a student’s first exposure to musical sight reading, so naturally students at this level find large quantities of practice material useful. A wide variety of practice exercises is also useful at other early grade levels as students come to grips with sight reading techniques. Students studying later levels–typically Grade 5 and above–often require fewer practice exercises. There are likely two reasons for this. Firstly, students at this level should already have a solid foundation of sight reading skills, and thus require less practice material. Secondly, at these levels students can use entire pieces from lower grades as sight reading exercises, reducing the need for purpose written material.

As discussed in Section 2.1, the set of individual expert-written sight reading exercises extracted from published exercise books were transformed into a set of expert models, where each model corresponds to one published exercise. The reason for modeling exercises individually rather than as a collective is twofold. Firstly, targeting only the most typical characteristics of a group of exercises ignores the variety in musical and technical content in exercises of even the lowest level of difficulty. This severely limits the variety of solutions the algorithm can generate, as it would be constrained by a single expert model for any one difficulty level. The second reason relates to the purpose of the experimental design in exploring the capabilities of the algorithm. If the algorithm is tasked only with generating ‘typical’ sight reading exercises, the results only indicate how it performs generating typical sight reading exercises. To gain a wider understanding of the algorithm’s performance, it should be tested on a broader range of input. Using models based on individual expert-written examples allows this to be done.

#### 2.4.2 Algorithm Configurations

Given a configuration, the algorithm generates tree structures using an evolutionary approach, as described in Section 2. When the algorithm is finished the best candidate is converted into a text format supported by music21^a^, which is then used to render the solution and save it in the MusicXML format.

A unique algorithm configuration was created for each expert model. Each of these configurations (i.e., parameter sets) covers three elements:

- Fixed characteristics,
- target characteristics, and
- evolutionary parameters.

The fixed characteristics represent targets for the algorithm which can be set or ‘hard-coded’ during initialization. These characteristics are:

- Length (number of bars)
- time signature
- key signature
- range
- use of ties, and
- use of rests.

There are two reasons for setting these characteristics as hard limits rather than targets that may or may not be achieved. Firstly, as discussed in Section 2.2.2, both the length and time signature are required to form the structure of the melody tree used to represent candidate solutions. Given this, they need to be fixed during initialization, and can not change during the evolutionary process without requiring fundamental alternations to the melody tree structure.

The second reason relates to the key signature, range, use of ties, and use of rests, in that setting non-negotiable limits on these characteristics significantly reduces the search space the algorithm needs to explore. If the key signature and range is known, random pitch selection can be restricted to a pool of pitches which are both in the given key signature and within the given range. As pitches outside the target range and key signature add no value to a candidate solution, this approach serves only to reduce the search space–it is highly unlikely to reduce the quality of the final solution, only the time needed to find it.

Similarly, if the melody of the expert model being used does not include any rests or ties, then rests and ties should not be introduced into the solution space. That is, mutations relating to rests and ties should not be applied, and the initial population of candidate solutions should not contain any rests or ties.

The target characteristics relate to specifying target values for each of the six fitness measures defined in Section 2.3.2. Unlike the fixed characteristics, these targets may not be perfectly met. Targets for the *target note lengths*, *target rest lengths*, *target intervals*, and *melody shape* measures can be found directly within an expert model. Targets for the remaining two measures–*allowable lengths* and *allowable intervals* can be inferred from these values. This was discussed in Section 2.3.2.

Finally, the evolutionary parameters relate to values which influence the evolutionary process but which are not specific to the chosen application of generating musical sight reading exercises. These remain static regardless of the expert model being used:

- Population size: 50
- Number of elites: 1
- Probability of mutation: 0.01 (i.e., 1%)
- Random function: Gaussian
- Selection method: Pareto
- Termination criteria: 100 generations with no improvement in the best candidate

Each configuration is also assigned a fixed random number generator seed, so that its output can be reproduced.

The parameters of the algorithm are intended to be specific enough to guide the evolutionary process, but still broad enough that there are many acceptable solutions for a given expert model. To show this, three configurations were created for each expert model, differing only in their random number generator seed. This means that after being executed with every configuration the algorithm will have generated three different sight reading exercises for each expert model. Comparing these results will indicate how consistent the algorithm is in finding acceptable solutions, and the similarities between solutions generated using the same set of targets.

#### 2.4.3 Evaluating the Generated Exercises

Describing exactly what makes a musical sight reading exercise fit for purpose requires quantifying both the esthetics of the melody as well as its technical appropriateness with respect to a specific difficulty level and instrument. Either one of these tasks is uniquely challenging on its own. Some guidance can be found by examining existing published exercises. These mostly reveal the technical properties which are appropriate for each difficulty level, such as the acceptable note lengths, interval sizes, and use of syncopation. Additional guidelines can be inferred. For example, some sequences of notes are clearly unplayable. Other components, such as repeated large intervals between notes, are widely accepted as being difficult to play (Schoenberg, 1967).

Other factors to consider relate to the esthetics of the melodies. Musical esthetics are notoriously difficult to quantify and remain the subject of much debate and ongoing research. One reason for this is that musical rules tend to be derived empirically, in that they emerge by examining trends in common practice rather than being determined *a priori*. Another reason is that they are largely contextual, depending on the culture, genre, age, and purpose of the music in question. This means that not only are they open for discussion and interpretation, but they also evolve over time.

Fortunately, the application domain of the algorithm presented in this work naturally restricts the scope of the musical rules that need to be considered. The purpose of the proposed algorithm is to generate sight reading exercises which would be suitable for students preparing for formal musical examinations such as those facilitated by the Australian Music Examinations Board (AMEB) and the United Kingdom’s Associated Board of the Royal Schools of Music (ABRSM). The curricula developed by these organizations strictly fall within Western Classical Music from the Common Practice period. This is a relatively well-documented period with a number of widely accepted musical guidelines for aesthetically pleasing melodic and harmonic structures. As the algorithm focuses on monophonic melodies, only guidelines relating to melodic structures need to be considered.

Additionally, sight reading exercises are uniformly short in length, meaning that complex concepts of musical form, which describe formal structures for musical pieces to follow, do not apply.

Each generated exercise was evaluated against a ruleset containing 29 rules relating to technical appropriateness and melodic esthetics. Evaluation was performed by one person who has over 20 years of musical experience, and who has obtained formal musical qualifications in both performance and musical theory studies. The proportion of an exercise in violation of the ruleset was translated to a Likert quality rating on a five-point scale according to Table 4. This means that although the weighting of the rules is equal, their impact on the final score for a melody differs as some are easier to violate than others. Exercises assigned a “Very good”, “Good”, or “Average” rating are said to be “fit for purpose”, with the remainder being ‘unfit for purpose’.

**TABLE 4:** Criteria for assigning each generated sight reading exercise a rating.

Rating	Fit for purpose?	Percentage of melody violating ruleset
Very good	Yes	−
Good	Yes	≤10%
Average	Yes	≤25%
Bad	No	>25%
Very bad	No	Unplayable elements

Each exercise is also given a rating based on whether it can be improved or upgraded with a small number of alterations. An exercise is said to be “improved” if it was already fit for purpose and becomes more so as a result of the changes. Alternatively, an exercise is said to be ‘upgraded’ if the changes transform it from being unfit to being fit for purpose. Currently, changes are made manually when assessing each exercise.

When determining potential changes, at most 5% of the melody can be altered. Acceptable alterations include changes to note pitches, note and rest lengths, and note and rest placements. Only changes which could be represented algorithmically should be used, as it is intended that these changes could be incorporated into the algorithm in the future. Once a small set of changes is made, an exercise is evaluated again with respect to the ruleset and a new Likert rating is assigned.

The post-alteration ratings serve a dual purpose. They show the potential of the algorithm to be improved, and serve to highlight the biggest problems currently preventing the generated exercises from being more fit for purpose. This information could be used to drive future work.

The evaluation ruleset was derived from an examination of relevant literature and published expert-written examples (i.e., expert models) of sight reading exercises. As well as relating to the technical appropriateness of an exercise, the melodic esthetics of an exercise, or both, each individual rule can be further categorized as relating to one of four facets:

- Note/Rest selection

Rules relating to the length, pitch, and location selected for each note and rest in the melody.

- Intervals

Rules relating to the size and placement of intervals.

- Melodic structure

Rules relating to the shape and form of the melody.

- Rhythmic structure

Rules relating to the sequences of note and rest lengths in the melody.

Many of the rules refer to the “strong” beats of a melody. Beats which are seen as “strong” depend on the time signature. In time signatures where bars can be divided into two equal parts, the first beat and the beat half-way through are strong (e.g., beats 1 and 3 in $\frac{4}{4}$ bars are strong). In all other time signatures the first beat of the bar is strong.

Some rules also refer to scale degrees, either numerically or in roman numerals. In this case, the scale degrees should be interpreted with respect to the target key signature of the generated exercise (e.g., scale degree 3 in C major would indicate the pitch E).

Many of the rules are written generally, for example “An exercise should only use rest lengths seen in expert models of the same difficulty level.” In order to implement the ruleset a reasonable sample of expert models need to be collected. For the application of the algorithm presented in this work these expert models are those for the flute described in Section 2.1. The ruleset can easily be translated to evaluate exercises for other monophonic instruments by replacing this set of expert models with another specific to the instrument being considered.

Table 5 summarizes the ruleset, including the source from which each rule was derived. The individual rules are defined as follows:

- Rest proportions

**TABLE 5:** The origin of each rule in the ruleset for evaluating algorithmically generated sight reading exercises. Note that there are no rules for evaluating just the melodic esthetics of rhythmic structures, only technical appropriateness alone or technical appropriateness and melodic esthetics combined.

Rules Evaluating…	Facet	Rule	Origin
*Technical*	*Note/Rest selection*	1. Rest proportions	Expert models
*Appropriateness*		2. Note lengths	Expert models
		3. Rest lengths	Expert models
		4. Tied notes	Expert models
	*Intervals*	5. Interval sizes	Expert models
		6. Interval proportions	Expert models; Miller (2005)
	*Melodic Structure*	7. Key signature	Expert models
	*Rhythmic Structure*	8. Time signature	Expert models
		9. Playability	Expert models
*Melodic*	*Note/Rest selection*	10. Note placement	Perricone (2000)
*Esthetics*		11. Tonic repetition	Laitz (2008); Perricone (2000)
		12. Opening note	Laitz (2008); Perricone (2000); Australian Music Examinations Board (2018b)
		13. Phrase endings	Expert models; Goetschius (2009)
		14. Peak note	Australian Music Examinations Board (2018b)
	*Intervals*	15. Tritones	Expert models; Perricone (2000); Goetschius (2009); Aldwell and Cadwallader (2018); Schoenberg (1967)
		16. Augmented and diminished intervals	Expert models; Perricone (2000); Goetschius (2009); Aldwell and Cadwallader (2018); Schoenberg (1967)
		17. Closing intervals	Expert models; Laitz (2008)
		18. Interval resolutions	Goetschius (2009)
	*Melodic structure*	19. Melodic direction	Goetschius (2009)
		20. Contextualizing leaps	Goetschius (2009); Perricone (2000); Kwalwasser (1955); Schoenberg (1967)
		21. Peak placement	Australian Music Examinations Board (2018b)
	*Rhythmic structure*	−	−
*Technical*	*Note/Rest selection*	22. Placement of long notes	Expert models; Goetschius (2009)
*Appropriateness*		23. Placement of rests	Expert models; Goetschius (2009)
*and Melodic*		24. Target key signature	Expert models; Miller (2005)
*Esthetics*	*Intervals*	25. Gap placement	Expert models; Laitz (2008); Miller (2005)
		26. Leaps	Laitz (2008)
	*Melodic structure*	27. Repetition	Expert models; Australian Music Examinations Board (2018b); Miller (2005)
		28. Length	Expert models; Goetschius (2009)
	*Rhythmic structure*	29. Syncopation	Expert models; Miller (2005)

No more than 10% of the melody should be made up of rests, unless a larger proportion is present in the target expert model.

- Note lengths

An exercise should only use note lengths seen in expert models of the same difficulty level. For example, Grade 1 exercises for the flute are expected to only use minim, crotchet, quaver, semiquaver, and semibreve length notes.

- Rest lengths

An exercise should only use rest lengths seen in expert models of the same difficulty level. For example, Grade 1 exercises for the flute are expected to only use crotchet, quaver, and semiquaver length rests.

- Tied notes
  Grade 1 exercises for the flute should not contain any tied notes.
  Grade 2 exercises for the flute should contain at most 5% tied notes.

If the target expert model contains a larger proportion of tied notes than those listed here, the maximum percentage of tied notes an exercise can contain is that of the target expert model.

- Interval sizes

An exercise should only use intervals seen in expert models of the same difficulty level. For example, Grade 1 exercises for the flute are expected to only use intervals up to a size of 7.

- Interval proportions

At least 90% of the intervals should be between 0 and 3 in size, inclusive (i.e., between a unison and a fourth).

At least 50% of the intervals should be between notes only one scale degree apart (i.e., a second).

7 Key signature

An exercise should only be written in a key signature seen in expert models of the same difficulty level. For example, Grade 1 exercises for the flute are expected to only be written in the keys of C, F, G, and B♭ major, or A, D, and E minor.

8 Time signature

An exercise should only be written in a time signature seen in expert models of the same difficulty level. For example, Grade 1 exercises for the flute are expected to only be written in $\frac{4}{4}$, $\frac{2}{4}$, or $\frac{3}{4}$.

9 Playability

Each bar of the melody should only contain sequences of note lengths seen in expert models of the same difficulty level. For example, a bar in a Grade 1 exercise for the flute in $\frac{4}{4}$ time can contain two minims in a row, but would not be filled with a string of semiquavers.

10 Note placement

Strong notes from the target key (i.e., 1, 3, 5) should be placed on at least 50% of the strong beats in the melody.

11 Tonic repetition

At least 10% of the strong beats in the melody should be filled with a tonic note.

12 Opening note

The opening pitch of an exercise should be 1, 3, or 5.

13 Phrase endings

The note before a rest should be at least crotchet length.

14 Peak note

The highest note in the melody should be used no more than 3 times.

15 Tritones

Tritones should never be used.

16 Augmented and diminished intervals

Augmented and diminished intervals should never be used.

17 Closing interval

The melody should end with 2 → 1, 7 → 1, 4 → 1, or 5 → 1.

18 Interval resolutions

After a jump (i.e., an interval greater than a fourth), instability should always be resolved.

- should resolve to 3.
- should resolve to 1 or 3.
- should resolve to 5.
- should resolve to 1.

19 Melodic direction

If the melody is moving up in pitch, it should not change direction on pitch 7.

If the melody is moving down in pitch, it should not change direction on pitches 4 or 6.

20 Contextualizing leaps

The melody should switch direction after a leap (i.e., an interval greater than a fourth).

If there are two leaps in a row, the first should be larger.

21 Peak placement

The peak should fall within the middle 50% of the melody.

22 Placement of long notes

80% of notes longer than a crotchet should be placed on strong beats of the bar.

23 Placement of rests

80% of rests should be placed on weak beats of the bar.

24 Target key signature
  All notes should have pitches from the target key signature.
25 Gap placement

There should be no more than 3 intervals of a third or more in sequence, unless the sequence forms an arpeggio.

26 Leaps

There should be no more than two intervals of a fourth or more in a row.

27 Repetition

A self-similar structure should not be repeated exactly more than twice in a row. If the structure is transposed when repeated, it is not considered to be repeated exactly.

28 Length

An exercise should be of a length, in bars, seen in expert models of the same difficulty level. For example, Grade 1 exercises for the flute are expected to only be 4, 8, 12, 14, or 16 bars long.

29 Syncopation
  Grade 1 exercises for the flute should contain no syncopation.
  Grade 2 exercises for the flute can contain up to 10% syncopation.

## 3 Results

### 3.1 Most Typical Characteristics

#### 3.1.1 Overview

A preliminary examination of the generated exercises can be done by comparing the most typical values for a set of measured characteristics between the expert-written and algorithmically-generated exercises. These results, presented in Sections 3.1.2 and 3.1.3, show how closely the generated exercises were able to match the characteristics of the expert-written exercises. This is a good indication of the fitness for purpose of the results.

#### 3.1.2 Grade 1

The Grade 1 generated exercises almost exactly match the most typical characteristics of the expert-written Grade 1 exercises, as seen in Table 6. Compared to the expert-written exercises, the generated exercises often exhibit a slightly smaller range, the most common highest note falling one full tone from G5 to F5. The generated exercises also include some semibreves, which were not seen in the expert-written exercises. They also exhibit slightly fewer crotchet rests and slightly increased proportions of quaver and semiquaver rests.

**TABLE 6:** Typical characteristics of expert-written and generated Grade 1 sight reading exercises. Ratios and proportions are represented in terms of time. Differences between the expert-written and generated exercises are highlighted in bold. Characteristics marked with ‘*’ are fixed and not expected to change.

Characteristic	Typical Value(s)	
	Expert-written exercises	Generated exercises
Key signature*	F major, C major	F major, C major
Time signature*	$\frac{4}{4}$	$\frac{4}{4}$
Exercise length*	8 bars	8 bars
Range	14 semitones (one octave and one tone)	12 semitones (one octave)
	F4 → G5	F4 → **F5**
Note lengths	90–100% crotchets	90–100% crotchets
	0–10% quavers	0–10% quavers
	0–5% minims, dotted minims, semiquavers	0–5% minims, dotted minims, semiquavers, **semibreves**
Rest lengths	95–100% crotchets	**90**–100% crotchets
	0–5% quavers, semiquavers	0–**10**% quavers, semiquavers
Ratio of notes to rests	90% notes: 10% rests	90% notes: 10% rests
Intervals	95–100% gaps of 1 scale degree	95–100% gaps of 1 scale degree
(As scale degrees)	0–5% gaps of 0 or 2–7 scale degrees	0–5% gaps of 0 or 2–7 scale degrees

#### 3.1.3 Grade 2

Table 7 shows that, compared to the expert-written exercises, the generated Grade 2 exercises exhibit only slight differences in their most typical characteristics. As with the Grade 1 exercises, the most common highest note drops a full tone, this time from A5 to G5. The typical proportions of crotchet note lengths increase in range from 80–90% to 75–90%. This is compensated for by increases in other note lengths, but not enough to alter the most typical proportions. The typical proportions of rest lengths also change. Crotchets are used less frequently, a drop which is compensated for by an increased use of quavers, semiquavers, and minims.

**TABLE 7:** Typical characteristics of expert-written and generated Grade 2 sight reading exercises. Ratios and proportions are represented in terms of time. Differences between the expert-written and generated exercises are highlighted in bold. Characteristics marked with ‘*’ are fixed and not expected to change.

Characteristic	Typical Value(s)	
	Expert-written exercises	Generated exercises
Key signature*	G major, A minor, F major	G major, A minor, F major
Time signature*	$\frac{4}{4}\frac{3}{4}$	$\frac{4}{4}\frac{3}{4}$
Exercise length*	8 bars	8 bars
Range	12 semitones (one octave and one tone)	12 semitones (one octave)
	G4 → A5	G4 → **G5**
Note lengths	80–90% crotchets	**75**–90% crotchets
	0–10% quavers	0–10% quavers
	0–10% minims	0–10% minims
	0–5% dotted minims, semiquavers, dotted	0–5% dotted minims, semiquavers, dotted
	Crotchets, dotted quavers, semibreves	Crotchets, dotted quavers, semibreves
Rest lengths	95–100% crotchets	85–100% crotchets
	0–5% quavers, semiquavers, minims	0–**15**% quavers, semiquavers, minims
Ratio of notes to rests	95% notes: 5% rests	95% notes: 5% rests
Intervals	85–95% gaps of 1 scale degree	85–95% gaps of 1 scale degree
(As scale degrees)	0–10% gaps of 0 or 2 scale degrees	0–10% gaps of 0 or 2 scale degrees
	0–5% gaps of 2–7 scale degrees	0–5% gaps of 2–7 scale degrees

### 3.2 Target Characteristics

#### 3.2.1 Pitch Range

Compared to the expert-written exercises, the generated exercises exhibit a greater variety of ranges and an increased use of smaller ranges (i.e., ranges less than 12 semitones in size). However, overall, the pitch ranges of the expert-written and algorithmically-generated exercises are similar.

Although the range for each exercise was fixed with respect to a particular expert model, the algorithm does not enforce that the specified range be used to its limits, only that all selected pitches must fall within that range. It is for this reason that the range sizes and spreads differ between the expert-written and algorithmically-generated exercises.

#### 3.2.2 Proportion of Notes vs. Rests

The amount of time in each exercise that should be filled by notes and rests is not specified exactly in the expert models, but can be inferred from the target proportions of specific note and rest lengths. That is, if the target proportion for crotchet notes is 0.5, the target proportion of quaver notes is 0.25, and target proportion of crotchet rests is 0.25, it can be inferred that the target proportion of notes is 0.75.

In the generated exercises there were no cases where an exercise contained rests if its corresponding expert model contained no rests. This is because, as discussed in Section 2.4.2, rests were not introduced at any stage of the algorithm’s execution if there were no rests in the expert model used to derive the algorithm parameters. For cases where both note and rest target proportions were provided, the generated exercises regularly matched the given target proportions exactly.

#### 3.2.3 Note Lengths

The generated exercises closely emulate the target note lengths extracted from the expert models. However, at both the Grade 1 and 2 difficulty levels there were note lengths in some generated exercises that were not present in any of the expert-written exercises. For Grade 1 the only unallowable note length used was a semibreve. This is not of significant concern given that semibreves are valid note lengths, and not unheard of at a Grade 1 level even though they are not present in the sample of expert-written Grade 1 exercises used in this work. The unallowable note lengths at the Grade 2 level, however, represent more of an issue. These were notes such as doubly dotted quavers and hemidemisemiquavers, which are rarely if ever seen at even the highest difficulty levels. However, very few generated exercises contained such note lengths.

#### 3.2.4 Rest Lengths

As with the note lengths, the proportions of rest lengths in the generated and expert-written exercises are reasonably close.

However, at both difficulty levels the generated exercises exhibited some rest lengths that were not present in the expert-written exercises. For example, some Grade 1 generated exercises contained minim rests, which were not in any of the expert-written Grade 1 exercises. Some exercises also contained rests with lengths that would rarely be seen at any difficulty level, such as doubly dotted semiquavers.

Overall, the proportions of rest lengths are more variable in the generated exercises compared to the expert-written exercises. This indicates that the generated exercises were not always able to match the target rest proportions exactly. They were, however, able to come close. Additionally, the spread of proportional values in the generated exercises is close to those of the expert-written exercises.

The generated exercises were not able to as closely match the target rest lengths as they were the target note lengths. It is important to note that this is most likely a side effect of the exercises containing significantly fewer rests than notes. A low number of rests within each exercise means that discrepancies between the actual and target rest proportions are amplified simply because each individual rest represents a relatively large proportion of the overall rest time. This results in cases where a single rest length being of an “unallowable” length can have a large effect on the overall rest proportions. For example, if an exercise was given a target of containing 4 crotchet rests, having one of those rests generated as two quaver rests means that 25% of the rest time is filled by an “unallowable” length, and the target rest proportion was only 75% met. This situation is much less likely to happen with note proportions, simply because significantly more time within each exercise is filled by notes.

#### 3.2.5 Intervals

At each of the difficulty levels, the proportions of intervals exhibited in the generated exercises closely match the target proportions set by the expert models.

In both grade levels the use of intervals with a size of 0 is higher in the generated exercises than in the expert-written exercises. However, as this increase in use is only slight it is not overly concerning. Compared to the expert-written exercises, the generated exercises exhibit greater variation in interval proportions. As with the rest length proportions, this is most likely an indication that the generated exercises were not always able to exactly match the target proportions. They were, however, able to come close.

### 3.3 Fitness of the Generated Exercises

As shown in Table 8, the generated exercises consistently achieved high fitness values on every fitness measure. At each grade level there was at least one exercise that achieved a perfect score on each of the fitness measures. The average fitness value for each measure was consistently high, ranging between 0.95 and 0.99 inclusive. Similarly, the standard deviations of the fitness values were consistently low, indicating that little variance was exhibited across the fitness values for each measure. The minimum values are more variable, both across fitness measures and within the same fitness measure across different grade levels. However, given the high average fitness and low standard deviations, such minimum values represent outliers rather than trends.

**TABLE 8:** Summary of fitness values for each grade level of generated exercises. Shows that at least one exercise reached the maximum value for each fitness measure (i.e., 1.0), and that the average fitness values for each measure were high at every difficulty level.

Fitness measure	Grade	Minimum	Maximum	Average	SD
Target note lengths	1	0.53	1.0	0.95	0.11
	2	0.66	1.0	0.96	0.08
Target rest lengths	1	0.96	1.0	0.95	0.01
	2	0.94	1.0	0.99	0.01
Allowable lengths	1	0.65	1.0	0.99	0.04
	2	0.95	1.0	0.99	0.01
Target intervals	1	0.82	1.0	0.95	0.05
	2	0.92	1.0	0.97	0.03
Allowable intervals	1	0.94	1.0	0.99	0.01
	2	0.96	1.0	0.99	0.01
Melody shape	1	0.8	1.0	0.99	0.02
	2	0.88	1.0	0.99	0.01

^a^ http://web.mit.edu/music21/.

Overall, there are no fitness measures on which the generated exercises scored consistently better or worse. This is true both when comparing different fitness measures within a grade level, and when comparing fitness values on the same measures across different grade levels.

Given the application domain, it is likely that some of the fitness measures may have conflicting goals. That is, an increase in one fitness value may be directly related to a decrease in another. For example, consider an exercise which meets its target proportion of notes and rests, but contains two unallowable rest lengths (e.g., two quaver rests instead of a single crotchet rest). If one or both of those unallowable rests were changed to notes, the “Allowable lengths” fitness of that exercise would improve. However, the extra notes would mean that the exercise no longer meets the target note and rest proportions, thus decreasing its fitness in the “Target note lengths” and “Target rest lengths” measures. The set of results presented in this work indicate that the fitness measures do not influence one another, given that the generated exercises achieved consistently high fitness scores. As such, potentially conflicting goals among the fitness measures can be considered not to be an issue.

### 3.4 Fitness for Purpose of the Generated Exercises

#### 3.4.1 Grade 1

The majority of the Grade 1 generated exercises are “fit for purpose”, with over 60% being assigned a “Very good”, “Good”, or “Average” rating. This proportion increases to almost 80% when small repairs are made to some exercises.

**FIGURE 6:**
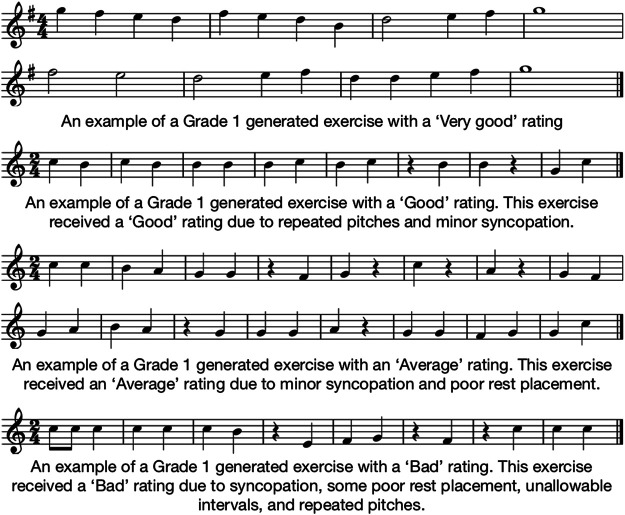
Examples of Grade 1 generated exercises assigned each of the Likert quality ratings. An example of an exercise rated as “Very bad” is not provided as none of the Grade 1 exercises were assigned this rating.

Figure 6 provides examples of generated Grade 1 exercises assigned each of the Likert ratings. None of the exercises at this difficulty level were assigned a “Very bad” rating, so no example has been given. Reasons are provided for each example’s rating.

#### 3.4.2 Grade 2

Approximately half of the Grade 2 generated exercises are fit for purpose. This is roughly 15% less than the Grade 1 generated exercises. The proportion of Grade 2 exercises rated as “fit for purpose” increases once small repairs are made, reaching approximately the same percentage as the Grade 1 generated exercises before repairs (i.e., roughly 60%). This drop in the number of “fit for purpose” exercises is an expected result due to the increased complexity of the Grade 2 exercises compared to Grade 1.

**FIGURE 7:**
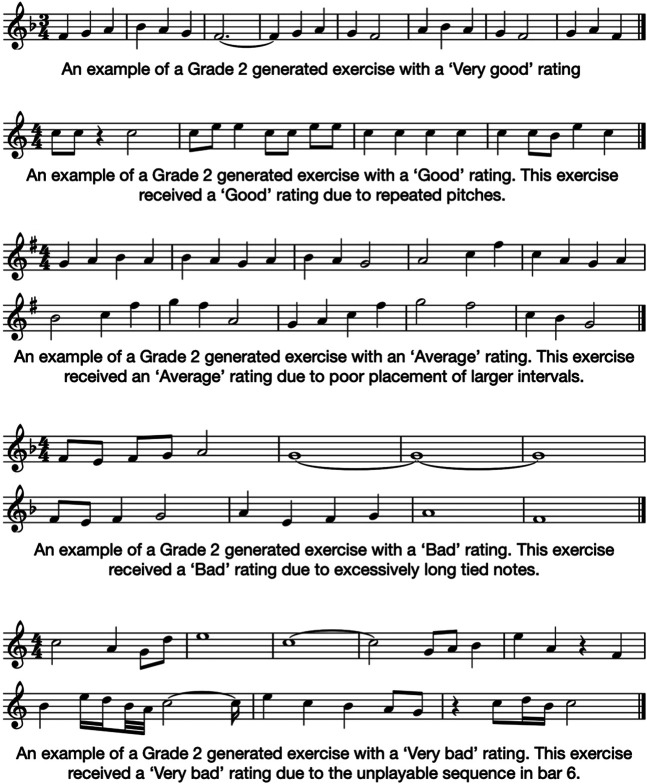
Examples of Grade 2 generated exercises assigned each of the Likert quality ratings. Note that the “Very bad” example was given this rating due to the rhythm in bar 6 being uncharacteristically difficult for the Grade 2 difficulty level.

The major difference between the Grade 1 and 2 ratings is the presence of “Very bad” exercises. These account for around 10% of the Grade 2 generated exercises, a proportion which does not change after repairs are made. This indicates that the “Very bad” exercises can not be upgraded in fitness for purpose, or even improved to a “Bad” rating. Given that the criteria for a “Very bad” rating is unplayable elements within an exercise, it is not surprising that a small number of changes could not resolve these issues. For reference, examples of generated Grade 2 exercises assigned each Likert rating are provided in Figure 7.

## 4 Discussion and Future Work

### 4.1 Current Capabilities of the Evolutionary Algorithm

These results show that the proposed EA is capable of emulating the characteristics of expert-written sight reading exercises at the Grade 1 and 2 difficulty levels, and that it is able to do so in a way that is generally fit for purpose. This is a particularly promising outcome given the relatively simplistic and general nature of the fitness measures. The capabilities of the algorithm to produce appropriate sight reading practice material is additionally supported by the Likert quality ratings, which show that the generated exercises also conform to the expectations of musical esthetics and technical appropriateness defined by the field of music theory.

Although the results indicate the potential of the algorithm, there are areas in which the algorithm’s capabilities can be improved. When examining the Likert quality ratings of an exercise with respect to its characteristics there appear to be no links. That is, the quality of an algorithmically generated exercise is not related to its key signature, time signature, length, note proportions, or interval proportions. This indicates that the primary difficulty in applying the algorithm to generate exercises at higher difficulty levels will be in managing the overall increase in musical and technical complexity. Even at the difficulty levels currently examined (i.e., Grades 1 and 2), higher quality results should be possible were this complexity to be better modeled and incorporated into the evolutionary process.

For example, the presence of rests in an exercise affects its overall structure. If not placed carefully within a sequence of notes, rests can cause unintended syncopation or awkward breaks in phrasing. The existence of a rest also affects the measurement of intervals within an exercise, as two notes separated by a rest are not considered to be part of an interval during fitness calculations. Given that rests become more frequent in number and length at later difficulties, these issues will become more prominent.

The pitch range of exercises also grows with the difficulty level. For example, exercises at the Grade 2 level cover a greater range of pitches than exercises at the Grade 1 level. An increase in pitch range increases the solution space. This is because there are simply more potential pitches to select, thus more potential for an algorithm to select pitch sequences which are aesthetically or technically inappropriate. Naturally, this increase in the size of the solution space also causes an increase in the difficulty of algorithmically generating fit for purpose solutions. This problem is compounded when considering other musical artifacts, as the size of the solution space similarly increases with additions to the sets of allowable note lengths and intervals. The interaction of these elements also needs to be considered. For example, increasing the allowable intervals in an exercise where only crotchet note lengths are allowed would increase the overall solution space. If additional note lengths were also to be allowed, the solution space would increase exponentially, not linearly. This is because some interval sequences, which would have been appropriate between crotchets, would not be appropriate with shorter note lengths.

These issues were expected, particularly given the general approach taken to measuring fitness in this work. Further efforts in modeling the requirements of higher difficulty exercises and incorporating those models into the algorithm would help to manage the expanding size of the solution space, thus the algorithm’s capacity to generate more complex exercises.

One potential drawback of the Likert ratings currently presented in the results is that they were all measured using a single rater. To check for consistency more robustly it would be ideal to have the same exercises rated using the same framework by multiple experts.

### 4.2 Future Directions for Algorithmic Development

When developing an algorithm for generating musical sight reading exercises, there are trade-offs to be made. One key decision is whether the algorithm will be specific or general. For example, a specific algorithm might only generate Grade 2 exercises focusing on breath control for the clarinet. Alternatively, a general algorithm might aim to generate exercises for any wind instrument at any difficulty level. There are benefits and drawbacks to each approach. The more specific the target, the more focused the algorithm can be. This means more domain knowledge can be incorporated and more restrictive parameters can be set. It is likely that a specific approach would enable the quality of output to be improved. However, a specific approach would, by definition, also be limited in its utility. A general approach would need to consider many more factors. For example, for an algorithm to target multiple instruments it would need to model the differences between the technical requirements for those instruments. A more general algorithm is likely to have an increased utility. However, it also takes on the risk of attempting to cover too much scope, which would limit its ability to generate quality output.

The approach taken in this work is somewhere in between. It is not so general as to target many difficulty levels, but it also isn’t restricted to a single type of exercise. Although the application of the algorithm presented in this work was generating musical sight reading exercises for the flute, its parameters are purely data driven–they are not instrument-specific. Instead, appropriate values can be extracted from models of expert-written examples, which may relate to any monophonic instrument. This is discussed further in Section 4.4.

Some avenues for future development can be found in the ruleset used to evaluate the fitness for purpose of solutions. For example, the rule for ‘Note placement’ states that *Strong notes from the target key (i.e., I, III, V) should be placed on at least 50% of the strong beats in the melody*. This indicates that music sounds better when strong notes from the target key are placed on strong beats of the bar. Such note placements could be encouraged by the evolutionary algorithm. Doing so might reduce or even remove the need for this evaluation rule, but should also increase the esthetics of results by reinforcing the key signature.

A similar approach could be taken to reinforce the time signature of an exercise. This relates to the “Placement of long notes” rule, which states that *80% of notes longer than a crotchet should be placed on strong beats of the bar*, and the “Placement of rests” rule, which states that *80% of rests should be placed on weak beats of the bar*. By encouraging these optimal note and rest placements the music should ‘feel’ like it is written in the target time signature. This would increase the overall esthetics of the generated melodies and avoid some situations where phrases seem to end abruptly.

As an addition to modeling expert-written examples, the algorithm could incorporate alternative models of musical complexity. These models would be relative to a specific instrument. One possible model is the *musiplectics* system (Holder et al., 2015). In this work, Holder et al. (2015) defines a method for computationally measuring the complexity of a musical score for any instrument. Measuring the complexity of a score first requires the definition of several parameters for the chosen instrument. Currently, only the parameters for a B♭ clarinet are provided. Implementing support for more instruments represents a non-trivial quantity of work, most of which requires input from an expert in the instrument in question. However, doing so might result in a valuable addition to the capabilities of the evolutionary algorithm.

Another area of improvement for the algorithm would be to use chord progressions. Currently, the algorithm generates exercises within a particular key signature. It does not, however, create melodies which follow chord progressions. For example, a common 4 bar chord progression is *I, IV*, *V*, *I*. If the exercise was in C major, this would mean that the 4 bars would be rooted in C major, F major, G major, and C major, in that order. Chord progressions give a melody a sense of movement and interest. Although not considered in this work, the expert-written exercises do use chord progressions. As such, it would make sense for the algorithm to do so as well.

One way chord progressions could be implemented would be to use the progression map proposed by Stephen (2017). This map defines transitions between chords which will sound aesthetically pleasing. This implementation would not require any changes to be made to the tree structure used to represent melodies. Although each bar would be rooted in a different key, the melody overall would still be in the one key signature. That is, even if the second bar in a C major melody might be written in F major, it would still only use pitches from the C major scale. The ruleset for evaluating exercises, however, would need to be updated. This is because some of the rules explicitly reference the target key. For example, the “Note placement” rule states that *Strong notes from the target key (i.e., I, III, V) should be placed on at least 50% of the strong beats in the melody*. If the generated melodies were to follow chord progressions, the “target key” part of this rule would need to be interpreted as referring to the chord of the bar, not of the overall melody.

Some of the expert-written exercises also change key completely as they progress, or contain accidentals outside of the key signature. Neither of these features is currently supported by the algorithm. Allowing additional accidentals is trivial, as it would simply require removing the restriction within the algorithm preventing it from selecting pitches outside the target key. However, this implementation would introduce significant complexity to the system, as the potential for selecting poor sounding pitch sequences would dramatically increase. A better implementation would allow non-key pitches to be selected, but restrict when that could occur.

Allowing for complete key changes is a more difficult task. Currently, the melody tree structure does not record the key signature of the melody it describes. As such, it also does not support the ability to record a change in key signature. This is not necessarily an issue, as information relating to the key signatures can be recorded elsewhere. The true difficulty lies in determining when a key change is appropriate, what the new key should be, and how to smoothly transition between the old and new keys. Such functionality would require significant changes to the algorithm. It would also require the key change to be noted in the output so that the melody can be interpreted correctly.

Another feature of the expert-written exercises not currently shown in the generated exercises is anacruses. An anacrusis is where a single bar is split into two parts which are placed at the beginning and end of a melody. This type of structure is not supported by the melody tree or the algorithm, and adding support would require significant work.

### 4.3 Building Better Models of Expert Knowledge

Given that the algorithm is designed to emulate expert models of musical sight reading exercises, it stands to reason that developing better expert models would improve the quality of its output. Currently, the expert models are a combination of simple characteristics (i.e., key signature, time signature) and statistical measures (i.e., note/rest/interval proportions). As such, there is significant scope for further development in this area.

One area for potential development relates to the analysis of co-occurring features. Currently, the note, rest, and interval proportions are treated separately. However, it is possible that there are some dependencies between these features that are not currently being captured. For example, it might be that larger intervals are more likely to be placed on longer notes, and smaller intervals on shorter notes. Finding these types of co-occurrences should be a relatively simple task. More difficult would be determining which co-occurring features are important to emulate, and how they should be implemented.

A similar area is that of sequence or pattern identification. The expert-written exercises, and music in general, exhibit many clear patterns. For example, often a dotted quaver will be followed by a semiquaver, or four semiquavers will be used in sequence. These types of patterns generally serve to reinforce the beats within the music and create a sense of rhythmic stability. Identifying the use of these patterns within the expert-written exercises, and incorporating them into the algorithm would serve to both better emulate the characteristics of the expert-written examples, and create more generally aesthetically pleasing results.

A complex area that has not yet been addressed either through the analysis of expert-written exercises or in the algorithm development is that many musical sight reading exercises are targeted to developing a specific skill. For example, some exercises for the flute contain a large proportion of long notes to encourage the development of breath control. Others might specifically use a series of arpeggios to reinforce scale structures.

Incorporating this type of information into the algorithm would be a significant undertaking. It would require extensive expert knowledge to identify the purposes of different musical sight reading exercises and describe how they have been written to address these purposes. Developing the ability to algorithmically generate similarly targeted exercises would be as, if not more, difficult. However, doing so would greatly increase the utility of the generated exercises, as they would be able to more specifically target the needs of different users.

### 4.4 Applying the Algorithm to Other Instruments

Although the use of the algorithm presented in this work was to generate musical sight reading exercises for the flute, the parameters are purely data driven. That is, they are instrument-agnostic. Given this, the algorithm can be applied to generate exercises for any monophonic instrument. This would involve curating a set of expert-written examples, and using those examples to determine appropriate parameters. Additional work would be required to support polyphonic instruments, particularly in the development of the tree structure used to represent melodies.

To validate the algorithm’s abilities in generating exercises for other instruments, the evaluation ruleset would need to be revisited. While the rules themselves are instrument-agnostic, their exact interpretation is sometimes relative to the analysis of expert-written examples. If the algorithm were applied to another instrument, those rules would need to be revised with respect to a new set of expert-written examples.

## Data Availability

The datasets generated for this study are available on request to the corresponding author.
